# Identification of Human Islet Amyloid Polypeptide as a BACE2 Substrate

**DOI:** 10.1371/journal.pone.0147254

**Published:** 2016-02-03

**Authors:** Ingrid C. Rulifson, Ping Cao, Li Miao, David Kopecky, Linda Huang, Ryan D. White, Kim Samayoa, Jonitha Gardner, Xiaosu Wu, Kui Chen, Trace Tsuruda, Oliver Homann, Helene Baribault, Harvey Yamane, Tim Carlson, Jed Wiltzius, Yang Li

**Affiliations:** 1 Amgen, Cardiometabolic Disorders, South San Francisco, California, United States of America; 2 Amgen, Molecular Structure and Characterization, South San Francisco, California, United States of America; 3 Amgen, Medicinal Chemistry, Thousand Oaks, California, United States of America; 4 Amgen, Medicinal Chemistry, Cambridge, Massachusetts, United States of America; 5 Amgen, Pathology, South San Francisco, California, United States of America; 6 Amgen, Discovery Technologies, Thousand Oaks, California, United States of America; 7 Amgen, Biologics, Thousand Oaks, California, United States of America; 8 Amgen, Genome Analysis Unit, South San Francisco, California, United States of America; 9 Amgen, Pharmacokinetics and Drug Metabolism, South San Francisco, California, United States of America; Baylor College of Medicine, UNITED STATES

## Abstract

Pancreatic amyloid formation by islet amyloid polypeptide (IAPP) is a hallmark pathological feature of type 2 diabetes. IAPP is stored in the secretory granules of pancreatic beta-cells and co-secreted with insulin to maintain glucose homeostasis. IAPP is innocuous under homeostatic conditions but imbalances in production or processing of IAPP may result in homodimer formation leading to the rapid production of cytotoxic oligomers and amyloid fibrils. The consequence is beta-cell dysfunction and the accumulation of proteinaceous plaques in and around pancreatic islets. Beta-site APP-cleaving enzyme 2, BACE2, is an aspartyl protease commonly associated with BACE1, a related homolog responsible for amyloid processing in the brain and strongly implicated in Alzheimer’s disease. Herein, we identify two distinct sites of the mature human IAPP sequence that are susceptible to BACE2-mediated proteolytic activity. The result of proteolysis is modulation of human IAPP fibrillation and human IAPP protein degradation. These results suggest a potential therapeutic role for BACE2 in type 2 diabetes-associated hyperamylinaemia.

## Introduction

In the early 1900s investigators Eugene Opie and Arthur Wright described instances of hyaline degeneration in the pancreatic “islands of Langerhans” [1, 2]. It was later determined that what they observed as hyaline deposits were actually proteinaceous amyloid plaques [3] and it was these findings that first helped distinguish the pathology of type 2 diabetes (T2D) from that of type 1 diabetes. Amyloid plaques are composed of aggregates of fibrillar islet amyloid polypeptide (IAPP; also known as amylin), a 37 amino acid hormone selectively expressed in pancreatic beta-cells. IAPP is stored as a prohormone precursor, ProIAPP, in the secretory granule of the beta-cell and co-processed and co-secreted with insulin in a 1:100 molar ratio [4, 5]. In response to glucose, insulin is secreted into the circulation, accompanied by IAPP which persists in the circulation at a higher ratio than insulin due to slower clearance [6]. Under normal conditions the role of IAPP is beneficial to maintaining blood glucose homeostasis [7]. The pH and calcium concentration within the secretory granule may be instrumental in maintaining IAPP solubility, but structural studies suggest polymerization of IAPP is prevented by the direct interaction of insulin [8, 9]. In circumstances of defective ProIAPP processing or hyperamylinaemia, the equilibrium between IAPP forming a heterodimer with insulin may shift towards IAPP homodimerization [8]. Homodimerization rapidly leads to the formation of oligomers and subsequent fibrillation, eventually resulting in plaques within and around the islets [5, 10]. Although debate surrounds the precise mechanism of IAPP-mediated beta-cell toxicity, it is well established that islet amyloidosis occurs in over 95% of T2D patients and is a profound contributor to the pathology of the disease [4, 10].

IAPP is synthesized as an 89 residue pre-pro-precursor that is cleaved to the 67 amino acid ProIAPP form. The subsequent conversion of ProIAPP to IAPP occurs in parallel to the biosynthesis of proinsulin into insulin, which occurs in response to glucose exposure [4]. The proconvertases, PC1/3 and PC2, are required for proper processing of both insulin and IAPP [4]. Although the basic processing of ProIAPP into mature IAPP is well defined, much remains to be learned about both the contribution of ProIAPP to amyloidogenecity, and the possibility of further modifications to both ProIAPP and IAPP that could modulate amyloidosis and prevent beta-cell loss.

Amyloidosis in the pancreatic islets of T2D patients resembles that of amyloidosis observed in the brain of Alzheimer’s disease (AD) patients [11]. In the brain, amyloid β (Aβ) is derived from amyloid precursor protein (APP) and is the major component of neuritic plaques in AD patients [12]. Beta-site APP-cleaving enzyme 1 (BACE1, also called Memapsin 2) has long been known to be a primary enzyme responsible for processing APP into Aβ and has been a leading target for AD therapeutic intervention [13].

Beta-site APP-cleaving enzyme 2 (BACE2, also called Memapsin 1), is a 49kD type 1 membrane-bound aspartyl protease homologue of BACE1. *In vitro*, BACE2 can cleave APP at the β-secretase cleavage site, similar to BACE1 [14, 15]. However, studies indicate BACE2 functions predominantly by cleaving Aβ at phenylalanines 19 and 20, referred to as the “theta” secretase site, thus resulting in fragmented peptides that cannot dimerize [16]. More recently, investigators extended the description of BACE2 to that of an avid Aβ-degrading protease, providing support for BACE2 as a therapeutic candidate for AD [17]. The premise that BACE2 may be protective against APP-mediated amyloid deposition is supported by the *in vivo* findings that mice double transgenic for APP and BACE2 do not exhibit increased Aβ concentration in the brain or any worsening of neurological conditions [18]. In contrast to Aβ though, no protease has been described so far that can cleave mature human IAPP and modulate amyloidogenecity.

In humans, BACE2 is expressed at low levels in the central nervous system but at higher levels in peripheral organs including the stomach, colon, arteries and pancreatic beta-cells [19–23]. In recent years investigators reported data showing BACE2 inhibition imparts a positive effect on beta-cells, including sustaining a capacity for beta-cell proliferation to compensate for insulin demands in diabetic mice [21, 22, 24]. However, the correlations between AD and T2D, the shared amyloidogeneic nature of Aβ and human IAPP, the reported degrading capacity of BACE2 on Aβ, and the co-localization of BACE2 and IAPP in beta-cells, led us to explore a positive role for BACE2 activity in islet amyloidosis. Thus, we questioned whether, similar to Aβ, human IAPP is a BACE2 substrate, if BACE2 can cleave human IAPP at distinct phenylalanine residues, and, in so doing, modulate islet amyloid fibrillation.

## Materials and Methods

### Reagents

All IAPP peptides were synthesized by CS Bio (Menlo Park, CA). Peptides were solubilized in 100% HFIP (Sigma) and diluted to 10μM in the appropriate buffer conditions immediately prior to use. The final concentration of HFIP was 1% for all samples. Recombinant human insulin (Sigma) and human proinsulin (R&D Systems) were both reconstituted in PBS and used at a final concentration of 17μM.

### Generation of recombinant BACE2 and BACE1 proteins

Human BACE2 was produced from the stable transfection of serum-free CHO-S cells (Life Technologies) with a plasmid containing a Puromycin resistance gene (pSLX235a:huBACE2-serine (1–460)::V5::6xHis). The stable pool was expanded in serum-free growth media and final production was completed in a Wave Bioreactor using in-house media. At harvest, the cells were removed by centrifugation. The media was concentrated and buffer exchanged into 20mM Tris, pH 8, 150mM NaCl, and 10mM imidazole. The concentrated media was loaded onto NiNTA (Qiagen) and step eluted with 20mM Tris, pH 8, 200mM imidazole, 150mM NaCl. BACE2 was concentrated and incubated with anti-V5 agarose (Sigma) overnight at 4°C rotating. BACE2 was eluted with 0.1M acetic acid and neutralized with 1M Tris pH 9.2. The pool was concentrated and loaded onto a Superdex-200 gel filtration column in 20mM Tris, pH 7, 300mM NaCl. The BACE2 pool was aliquoted, flash-frozen, and stored at -70°C. Human BACE1 was produced from the stable transfection of CHO AM-1 cells (PDSRα19:huBeta-Secretase(E46S)::6xHis). The resulting adherent cell line was expanded in DMEM growth media supplemented with 10% dialyzed FBS. Final production was done in Corning Cell Stacks in serum-free DMEM/Ham'sF12 supplemented with 10% DMSO. The media was concentrated and buffer exchanged into PBS. 5mM imidazole was added and sample was loaded onto a NiNTA (Qiagen) column. BACE1 was eluted with a 10–200mM imidazole gradient in 20 mM Tris, pH 8, 200mM NaCl. BACE1 was diluted 10x with 20mM tris, pH 7 and loaded onto an anion exchange column, Q-HP (GE). BACE1 was eluted with a 0–250mM NaCl gradient in 20mM Tris pH 7. The BACE1 pool was aliquoted, flash-frozen, and stored at -70°C. Enzyme purity was determined to be >95% for BACE1 and almost 100% for BACE2, as measured by reduced and non-reduced SDS-PAGE gel analysis.

### Mass spectrometry

Studies were performed using an ultrafleXtreme MALDI-TOF mass spectrometry (Bruker Corporation). Concentrations, buffers and pH were tested and optimized in preliminary studies. The final concentrations of the reagents were: peptide, 10μM; enzyme, 100nM, and insulin and proinsulin, 17μM. Samples were prepared by acidifying 10 μl of each sample with 1 μl of 5% formic acid. Each acidified sample was loaded on Ziptip C18 (EMD Millipore Corporation). After washing with 10μl of 0.1% TFA twice, samples were eluted with 1 μl of 50% ACN, 0.1% TFA solution onto MALDI plate where it was mixed with 1 μl MALDI matrix. 0.5 M THAP (2, 4, 6, trihydroxy acetophenone) in ethanol mixed with 0.1 M aqueous diammonium hydrogen citrate in a ratio of 2:1 was employed as matrix. MALDI instrument was operated as reflectron mode.

### Thioflavin T binding assays

Enzymes were first diluted to the indicated concentration in buffer (50mM NaOAc and 1M NaCl, pH 5) containing 20μM Thioflavin T (Sigma); 200μl per reaction. Immediately before the start of the run, 2μl of peptide, solubilized in 100% HFIP to 1mM, as described above, was added to the reaction, for a final concentration of 10μM peptide per reaction. All conditions were run in triplicate, in 96-well black, clear bottom plates (Costar), and performed for 400–800 minutes at 37°C on a Varioskan Flash Multimode Reader (Thermo Scientific), capturing a reading every 5 minutes; fluorometric wavelength settings: 450nm (Excitation) and 482nm (Emission). Fibril formation was determined based on increased fluorescence intensity (y-axis).

### Cell assays and protein analysis

Plasmid DNA was purchased from Origene (Rockville, MD). Human IAPP (RC215074), human BACE1 (RC209115), and human BACE2 (RC204860) contain a c-terminal Myc-DDK-tag; the human APP plasmid (RC226994) is untagged. HEK293 cells (ATCC) were cultured in Dulbecco’s Modification Eagle’s Medium (Mediatech) with 10% fetal bovine serum (FBS; HyClone). βTC3 cells (DSMZ, Germany) were grown in DMEM supplemented with 15% horse serum (HyClone) and 2.5% FBS. Cells were plated one day before transfection at 7.5 x10^4^ cells per well onto 6-well plates and grown in complete growth medium. Transfections were performed using Lipofectamine 2000 (Life Technologies); control plasmid pCMV DNA was used to equilibrate all samples to 4 μg per reaction. After 40 hours incubation at 37°C, cells were washed twice with PBS and then lysed with RIPA buffer and PMSF (Cell Signaling Technology) and protease inhibitors (Roche). Protein concentration was determined using a BCA Protein Assay Kit (Pierce).

Thirty μg of protein cell lysate was prepared in NuPAGE LDS sample buffer and reducing agent (Life Technologies), heated to 95°C for 5 minutes and then loaded onto a precast Novex Tris-Glycine 18% gel or a NuPAGE Bis-Tris 4–12% gel (Life Technologies). Western transfers were made onto PVDF (for IAPP bands) or a Nitrocellulose (for APP bands) membranes (Life Technologies) using an iBLOT transfer system (Life Technologies). Human IAPP, BACE2 and BACE1 proteins were detected using an anti-DDK monoclonal antibody (Origene). Also used were anti-hAPP 6E10 (Covance) and β-actin (Sigma). Myc-DDK tagged IAPP HEK293T lysate was used as positive control to identify the correct hIAPP bands (LC400146; Origene). Imaging and relative band intensity was performed and determined using an Odyssey CLx Infrared Imagaing System and Image Studio Lite software (LI-COR).

Global transcript expression in HEK293 cells was assessed by RNA sequencing (RNA-Seq). RNA-Seq was performed on a cDNA library prepared from total RNA (2 μg; RIN score > 9.5) HEK293 isolated using Mirvana miRNA RNA isolation kits (Ambion) with on-column DNase treatment. Total RNA quality and concentration was determined using Bioanalzyer (Agilent) and Nanodrop (ThermoScientific) instruments. cDNA was prepared using a modified protocol based on the Illumina TruSeq RNA Sample Preparation Kit (Illumina) and the published methods for strand-specific RNA-Seq [25, 26]. After size selection of libraries (Pippen Prep; SAGE Biosciences), dUTP-containing cDNA strands were destroyed by digestion of USER enzymes (New England Biolabs) followed by PCR enrichment for introduction of strand specificity. The enriched cDNA libraries were analyzed by Agilent Bioanalyzer and quantified by Quant-iT^TM^ Pico-Green assays (Life Technologies). RNA sequencing reads (Illumina HiSeq platform, 75 bp paired end sequencing) were aligned to Human genome build 37 and FPKM (Fragments per Kilobase per Million sequenced), values normalized by third quartile were determined using Array Suite software (Omicsoft) and in-house software.

### BACE1 and BACE2 enzymatic assays

The enzymatic activity of both BACE1 and BACE2 is determined by the enhancement of fluorescence intensity upon enzymatic cleavage of the FRET (fluorescence resonance energy transfer) substrate. The cleavage sequence of the substrate is derived from the literature [27], and a fluorophor and a quencher dye are attached to the Lys side chain at the termini of the substrate. The human recombinant BACE1 and BACE2 FRET assays were performed in 50 mM Acetate, pH 4.5 / 8% DMSO / 100 μM Genapol / 0.002% Brij-35 using the same FRET substrate in a Costar 96-well black polypropylene plate. In dose-response IC_50_ assays, 10 concentrations of each compound were made using 1:3 serial dilutions in DMSO and pre-incubated with the enzyme for 60 min at room temperature. Subsequently, the FRET substrate was added to initiate the reaction. After 60 min at room temperature, the reaction was stopped by the addition of un-titrated 0.1 M Tris Base to raise the pH above the enzyme active range. The fluorescence intensity of each well was measured on Safire II microplate reader (Tecan, Switzerland), and the IC_50_s were calculated by fitting normalized activity data with a 4-parameter non-linear regression equation via Screener software (Genedata AG, Switzerland).

### BACE2 inhibition in vivo studies

All animal experiments were approved by the Institutional Animal Care and Use Committee of Amgen and cared for in accordance to the *Guide for the Care and Use of Laboratory Animals*, *8*^*th*^ Edition [28]. Mice were housed in an air-conditioned room at 22±2°C with a 12 hour light; 12 hour darkness cycle (0600–1800 hours). Mice were group housed until the start of the study at which point they were randomized based on body weight and blood glucose measurements and then separated into single housing conditions. Animals had *ad libitum* access to pelleted feed 2020x diet (Harlan Teklad) or D12492 60% high fat diet (Research Diets) and water (reverse osmosis-purified) via automatic watering system. At termination, mice were euthanized following AAALAC Inc. guidelines, using CO_2_ inhalation followed by exsanguination as a secondary method.

BACE inhibitors, Compound J and Compound 15 [21, 29] were dissolved in 10% HPbCD in water, pH2.2 with HCl. 6 week old B6.V-Lep^ob^/J male mice (JAX) were dosed by oral gavage with 30mg/kg of Compound J or Compound 15, and plasma was collected at 0.5, 1, 2, 4, 6 and 25 hours post administration. For the chronic dosing study, 6 week old B6.V-Lep^ob^/J male mice (JAX) were dosed daily by oral gavage for 17 days with vehicle (10% HPbCD in water, pH 2.2 with HCl, n = 14), Compound J (30mg/kg, n = 13), and Compound 15 (30mg/kg, n = 13). Positive control mice received Exendin 4 (Bachem), 20 μg/kg in PBS, daily by intraperitoneal injection and concurrently received 10% HPbCD in water, pH 2.2 with HCl by oral gavage to mimic the inhibitor dosing regimen (n = 14). A glucose tolerance test was conducted on day 14. Mice were first fasted for 12 hours and then injected with a 10% glucose solution via intraperitoneal injection. Blood glucose levels were read at time 0 and then 15, 30 and 60 minutes post-injection. Mice were pulsed 24 hours before harvest by intraperitoneal injection with 5-Bromo-2’-deoxyuridine (BrdU, Sigma). On day 17 the study was terminated and pancreas tissue was collected, fixed in 10% neutral buffered formalin for 18 hours at 4°C, and processed for paraffin-embedding. For target coverage analysis, 10 week old B6.V-Lep^ob^/J male mice (JAX) were dosed by oral gavage with vehicle, Compound J (30mg/kg) or Compound 15 (30mg/kg), n = 2 per group. Eighteen hours later, islets from each mouse were harvested as described [30]. Lysates were prepared in RIPA buffer and samples were loaded on a 4–20% SDS-page TGX gel (BioRad). Antibodies: anti-mouse BACE2 (Santa Cruz Bio, SC-271212), anti-mouse Tmem27 (Abcam, ab-89058), and anti-β-actin (Sigma).

### Immunohistochemistry

*S*taining was performed on formalin fixed, paraffin embedded mouse pancreas cut at four microns and mounted on charged glass slides. Staining was done online using the Dako Autostainer (Dako Inc). The slides were pre-treated by a heat induced epitope retrieval method using an antigen retrieval buffer (Dako), followed by an avidin and biotin block (Vector Labs), and background block using Peroxidased 1 and Background Sniper (Biocare Medical) prior to antibody incubation. Slides were first stained for BrdU (Accurate Chemical), followed by a goat-anti-rat secondary step, followed by streptavidin-HRP, followed by a DAB chromogen (Dako) for detection of proliferating cells. Next, slides were stained for insulin (Dako), followed by a goat-anti-guinea pig secondary step, followed by an alkaline phosphatase step, followed by Vulcan Fast Red chromagen (Biocare Medical) for detection of beta-cells. All slides were counterstained with Modified Mayer’s Hematoxylin (DAKO). Morphometric analysis was performed using a Scan Scope XT (Aperio) and both Image Scope (Aperio) and Indica Lab (Indica Lab) software. For each animal, 5 sections, ≥ 300 microns apart, were evaluated.

## Results

### Differential cleavage of hIAPP by BACE2 and BACE1

**Fig 1:**
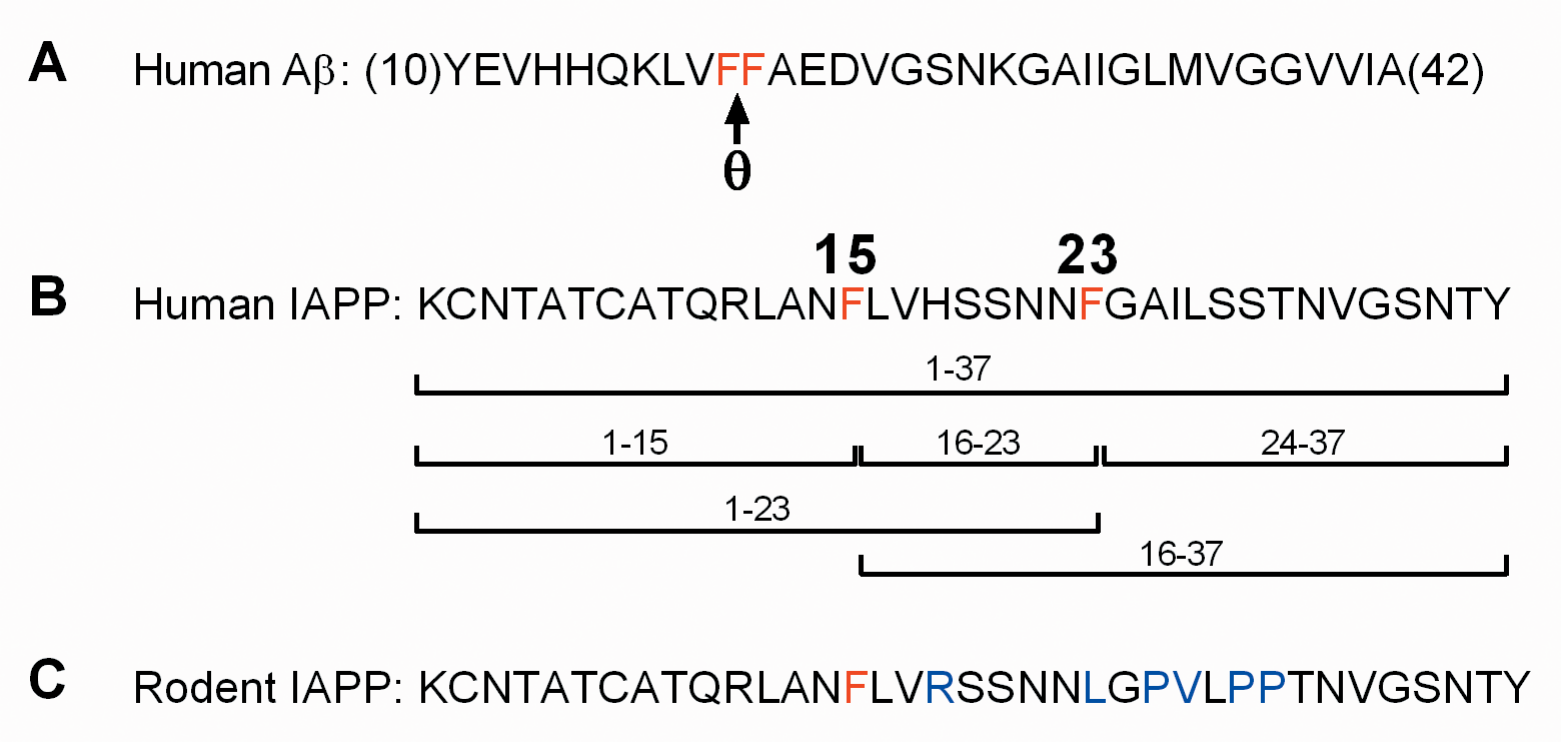
Comparison of human Aβ, human IAPP and rodent IAPP peptide sequences. (A) The amino acid sequence of human Aβ (10–42); the described “theta” site (θ) is indicated by an arrow between phenylalanine residues 19 and 20 (in red). (B) The amino acid sequence of mature human IAPP; phenylalanine residues at positions 15 and 23 (in red). The potential peptide fragment species induced by BACE2-mediated cleavage are indicated: 1–37, 1–15, 16–23, 24–37, 1–23, and 16–37. (C) The amino acid sequence of mature rodent IAPP; the phenylalanine residue at position 15 (red) and residue differences between human and rodent sequences (blue) are highlighted.

**Fig 2:**
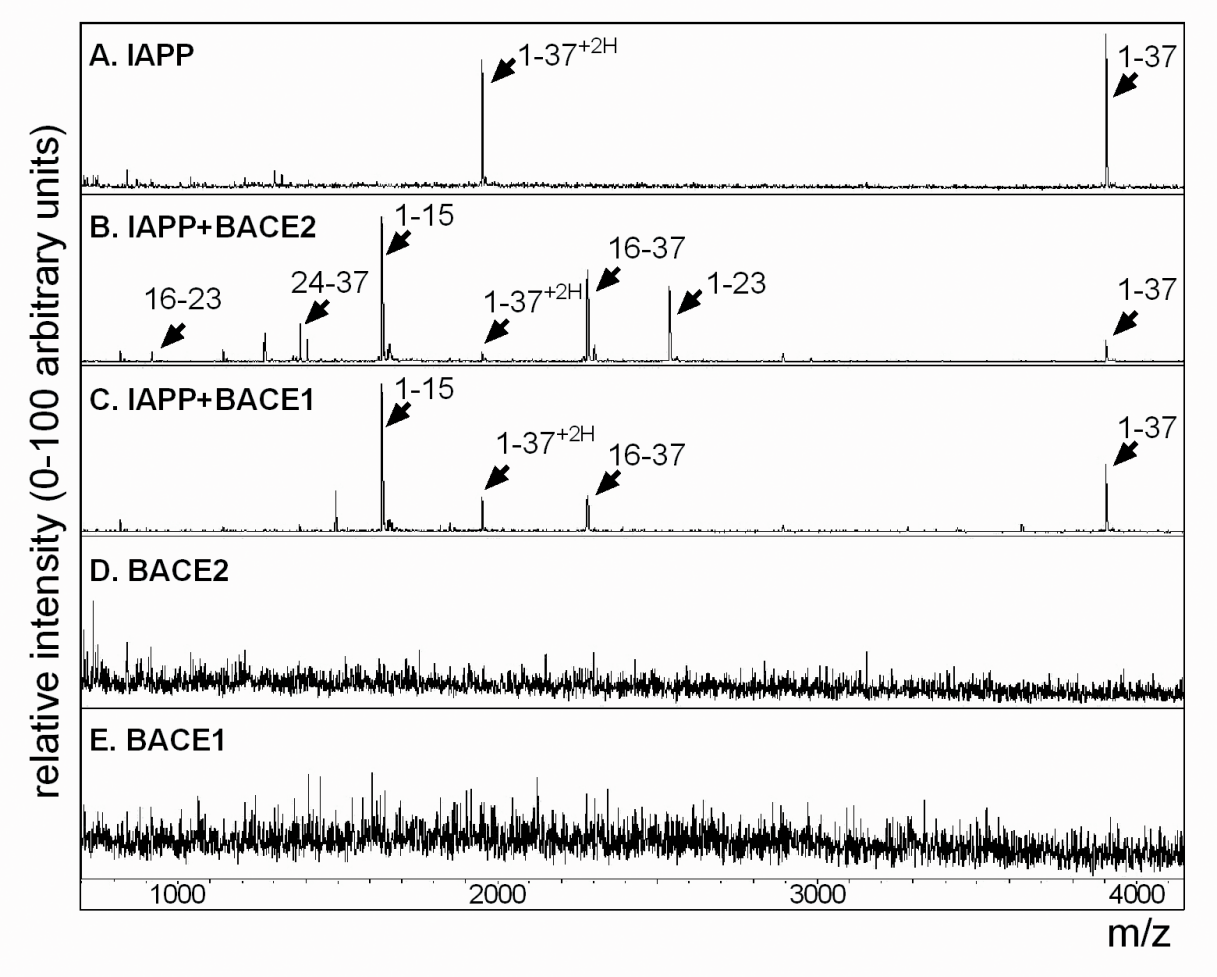
Mass spectrometry of hIAPP confirms proteolytic cleavage by recombinant BACE2 at F15 and F23. (A) MS analysis of hIAPP indicates a single protonated peak at m/z 3903 and a double protonated peak at m/z 1952. (B) Addition of recombinant BACE2 to hIAPP results in peaks at m/z 917; 16-LVHSSNNF-23, m/z 1382; 24-GAILSSTNVGSNTY-37, m/z 1639; 1-KCNTATCATQRLANF-15, m/z 2281; 16-LVHSSNNFGAILSSTNVGSNTY-37, and m/z 2537; 1-KCNTATCATQRLANFLVHSSNNF-23. (C) Addition of recombinant BACE1 indicates peaks only at m/z 1639 (1–15) and m/z 2281 (16–37), corresponding to cleavage only at F15. (D) Recombinant BACE2 alone and (E) recombinant BACE1 alone. All reactions were performed in 50mM NaOAc and 1M NaCl, pH 5 for 4 hours at 37°C. Peptide fragment sequences are detailed in Table 1.

To determine if recombinant human BACE2 is sufficient to proteolytically cleave human IAPP peptide (hIAPP), we used mass spectrometry (MS) to identify hIAPP fragments resulting from BACE2 digestion *in vitro*. Previous work identified BACE2-preferential proteolysis at the theta (θ) site of Aβ (Fig 1A), thus based on the presence of phenylalanine at the cleavage site in human Aβ, we hypothesized possible cleavage sites at residues F15 and F23 of mature hIAPP may result in a variety of processed peptide species (Fig 1B). Upon addition of recombinant BACE2 to hIAPP peptide, several new prominent peaks emerged in addition to the single protonated peak, m/z 3903, and double protonated peak, m/z 1952, of mature hIAPP peptide, KCNTATCATQRLANFLVHSSNNFGAILSSTNVGSNTY (compare Fig 2A to Fig 2B, and Table 1). The mass of the new peaks were consistent with proteolytic cleavage at F15 and F23, thereby creating the resulting fragments: 1–15; KCNTATCATQRLANF, 16–37; LVHSSNNFGAILSSTNVGSNTY, 1–23; KCNTATCATQRLANFLVHSSNNF, 16–23; LVHSSNNF, and 24–37; GAILSSTNVGSNTY (Figs 1B and 2B). When hIAPP was incubated with recombinant BACE1, however, only two new peaks, 1–15 and 16–37, emerged correlating to a single digestion at F15 (Fig 2C). Control conditions of recombinant BACE2 and BACE1 alone are shown (Fig 2D and 2E, respectively). Similar peak results were detected using commercial recombinant mouse BACE1 and BACE2 (data not shown). hIAPP digestion was more pronounced under acidic conditions (data not shown); which is both optimal for BACE2 and BACE1 enzymatic activity [31] and more analogous to the acidic environment of secretory vesicles [5].

**Table 1:** Key for human IAPP peptide sequences and cleaved fragments.

m/z	Description	Sequence
3903	hIAPP 1–37, +H	KCNTATCATQRLANFLVHSSNNFGAILSSTNVGSNTY
1952	hIAPP 1–37, +2H	KCNTATCATQRLANFLVHSSNNFGAILSSTNVGSNTY
917	hIAPP 16–23	LVHSSNNF
1382	hIAPP 24–37	GAILSSTNVGSNTY
1639	hIAPP 1–15	KCNTATCATQRLANF
2281	hIAPP 16–37	LVHSSNNFGAILSSTNVGSNTY
2537	hIAPP 1–23	KCNTATCATQRLANFLVHSSNNF

**Fig 3:**
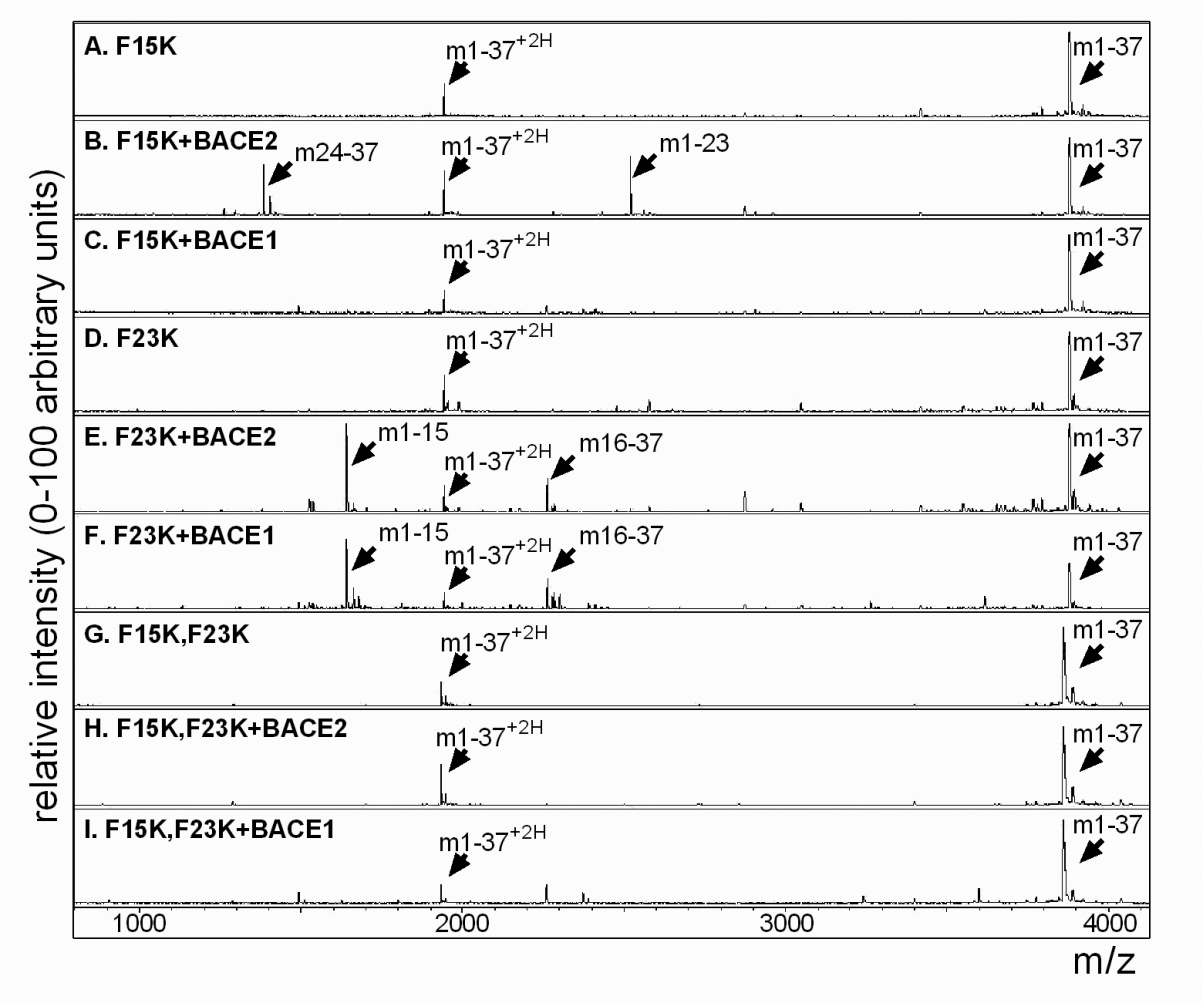
hIAPP mutants block predicted digestion by BACE2 and BACE1. (A) MS analysis of hIAPP F15K mutant peptide alone indicates a single protonated peak at m/z 3886 and a double protonated peak at m/z 1943. (B) Addition of recombinant BACE2 to mutant hIAPP F15K results in two new peaks at m/z 1382; 24-GAILSSTNVGSNTY-37, and m/z 2520; 1-KCNTATCATQRLANFLVHSSNNK-23. (C) Mutant hIAPP F15K is unchanged by the addition of recombinant BACE1. (D) Mutant hIAPP F23K. (E) Addition of recombinant BACE2 to mutant hIAPP F23K results in two new peaks at m/z 1639; 1-KCNTATCATQRLANK-15, and m/z 2263; 16-LVHSSNNFGAILSSTNVGSNTY-37. (F) Addition of recombinant BACE1 to mutant hIAPP F23K also results in two new peaks at m/z 1639; 1-KCNTATCATQRLANK-15, and m/z 2263; 16-LVHSSNNFGAILSSTNVGSNTY-37. (G) Double mutant, hIAPP F15K,F23K alone. Double mutant hIAPP F15K,F23K, is unchanged by the addition of recombinant BACE2 (H) or by the addition of recombinant BACE1 (I). All reactions were performed in 50mM NaOAc and 1M NaCl, pH 5 for 4 hours at 37°C. Peptide fragment sequences are detailed in Table 2.

To test the specificity of hIAPP digestion by BACE2 at F15 and F23, and BACE1 at F15, mutant hIAPP peptides were generated in which phenylalanine residues at positions 15 and 23 were substituted to lysines, as described previously [8] (Fig 3A, 3D and 3G and Table 2). We systematically tested the mutant peptides and confirmed a substitution of F15K blocks the generation of the 1–15 and 16–37 fragments by both BACE2 and BACE1 (Fig 3B and 3*C*), whereas F23K blocks the 24–37 and 1–23 fragments made by BACE2 (Fig 3E and 3F). As predicted, double F15K,F23K mutants inhibited all the proteolytic cleavage products of hIAPP by both BACE2 and BACE1 (Fig 3H and 3I).

**Table 2:** Key for mutant human IAPP peptide sequences and cleaved fragments.

m/z	Description	Sequence
3886	Mut-hIAPP F15K 1–37, +H	KCNTATCATQRLANKLVHSSNNFGAILSSTNVGSNTY
3886	Mut-hIAPP F23K 1–37, +H	KCNTATCATQRLANFLVHSSNNKGAILSSTNVGSNTY
3867	Mut-hIAPP F15,23K 1–37, +H	KCNTATCATQRLANKLVHSSNNKGAILSSTNVGSNTY
1943	Mut-hIAPP F15K 1–37, +2H	KCNTATCATQRLANKLVHSSNNFGAILSSTNVGSNTY
1943	Mut-hIAPP F23K 1–37, +2H	KCNTATCATQRLANFLVHSSNNKGAILSSTNVGSNTY
1933	Mut-hIAPP F15,23K 1–37, +2H	KCNTATCATQRLANKLVHSSNNKGAILSSTNVGSNTY
1382	Mut-hIAPP 24–37	GAILSSTNVGSNTY
1639	Mut-hIAPP 1–15	KCNTATCATQRLANK
2263	Mut-hIAPP 16–37	LVHSSNNKGAILSSTNVGSNTY
2520	Mut-hIAPP 1–23	KCNTATCATQRLANKLVHSSNNK

Amino acid F→K substitutions are underlined.

**Fig 4:**
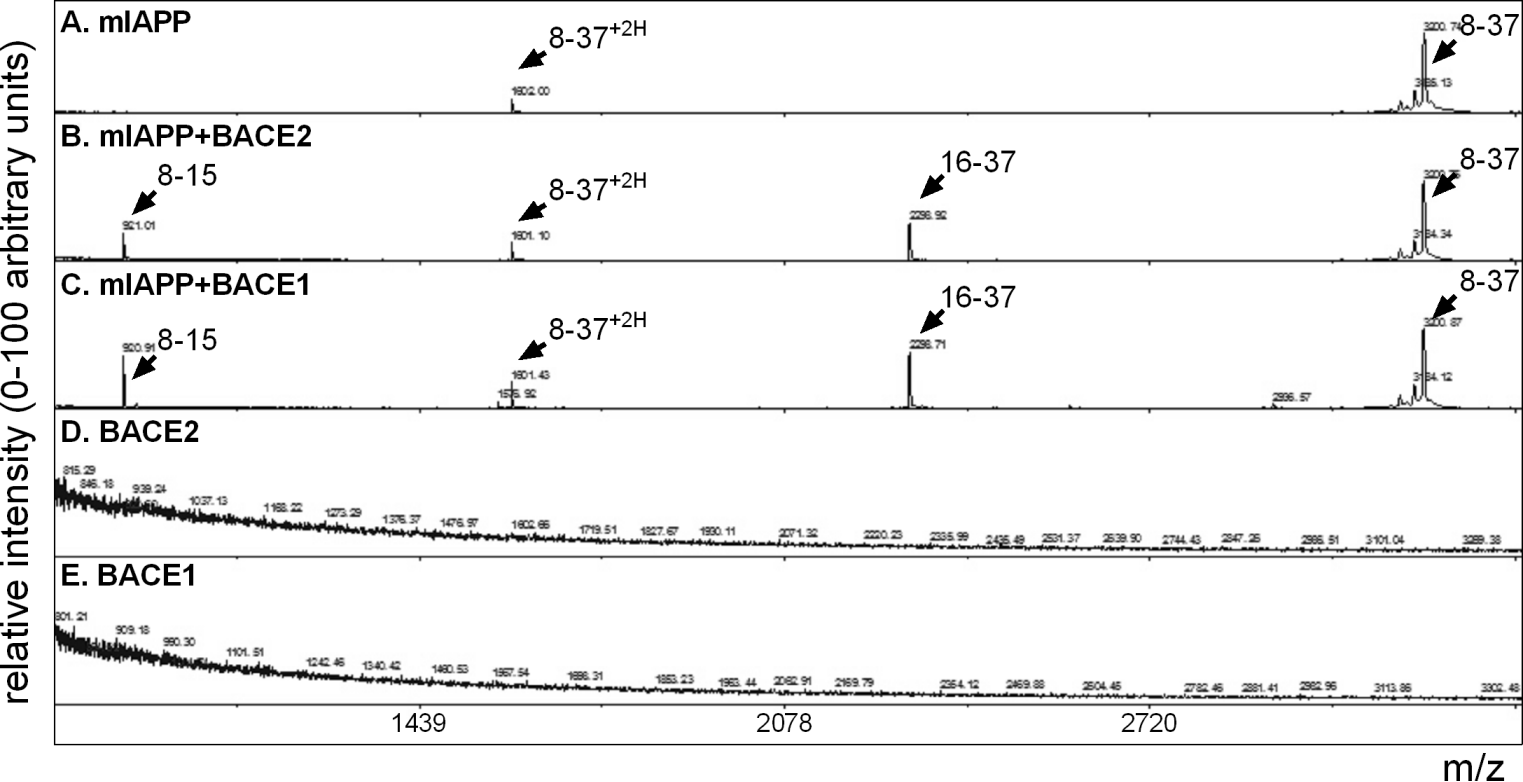
BACE2 only clips mIAPP at phenylalanine 15. (A) MS analysis of mIAPP peptide (8–37) indicates a single protonated peak at m/z 3200 and a double protonated peak at m/z 1602. (B) Addition of recombinant BACE2 to mIAPP peptide (8–37) results in two new peaks at m/z 920; 8-ATQRLANF-15, and m/z 2296; 16-LVRSSNNLGPVLPPTNVGSNTY-37. (C) Addition of recombinant BACE1 to mIAPP peptide (8–37) also results in two new peaks at m/z 920; 8-ATQRLANF-15, and m/z 2296; 16-LVRSSNNLGPVLPPTNVGSNTY-37. (D) Recombinant BACE2 alone and (E) recombinant BACE1 alone. All reactions were performed in 50mM NaOAc and 1M NaCl, pH 5 for 4 hours at 37°C. Peptide fragment sequences are detailed in Table 3.

The amino acid alignment of mature IAPP protein sequences from different species indicates a high degree of evolutionary conservation. There are only six residue differences between human and rodent IAPP (Fig 1B and 1C), however these changes, particularly between residues 20–29, are sufficient to render rodent IAPP non-amyloidogeneic [32–35]. Importantly, the phenylalanine at position 23 in the human sequence is replaced by a leucine in the mouse [5]. Accordingly, mouse IAPP, mIAPP; ATQRLANFLVRSSNNLGPVLPPTNVGSNTY (Fig 4A), incubated with either BACE2 (Fig 4B) or BACE1 (Fig 4C) led to cleavage only at F15, resulting in fragment 8–15; ATQRLANF, and fragment 16–37; LVRSSNNLGPVLPPTNVGSNTY (Table 3). This data further demonstrates hIAPP is a substrate for both BACE2 and BACE1, highlights residue F23 of hIAPP as a BACE2-specific cleavage site, and signifies the importance of proteolytic cleavage of hIAPP F23 by BACE2 in modulating fibril formation.

**Table 3:** Key for mouse IAPP peptide sequences and cleaved fragments.

m/z	Description	Sequence
3200	mIAPP 8–37, +H	ATQRLANFLVRSSNNLGPVLPPTNVGSNTY
1602	mIAPP 8–37, +2H	ATQRLANFLVRSSNNLGPVLPPTNVGSNTY
920	mIAPP 8–15	ATQRLANF
2296	mIAPP 16–37	LVRSSNNLGPVLPPTNVGSNTY

### Insulin blocks BACE2-mediated cleavage of human IAPP

**Fig 5:**
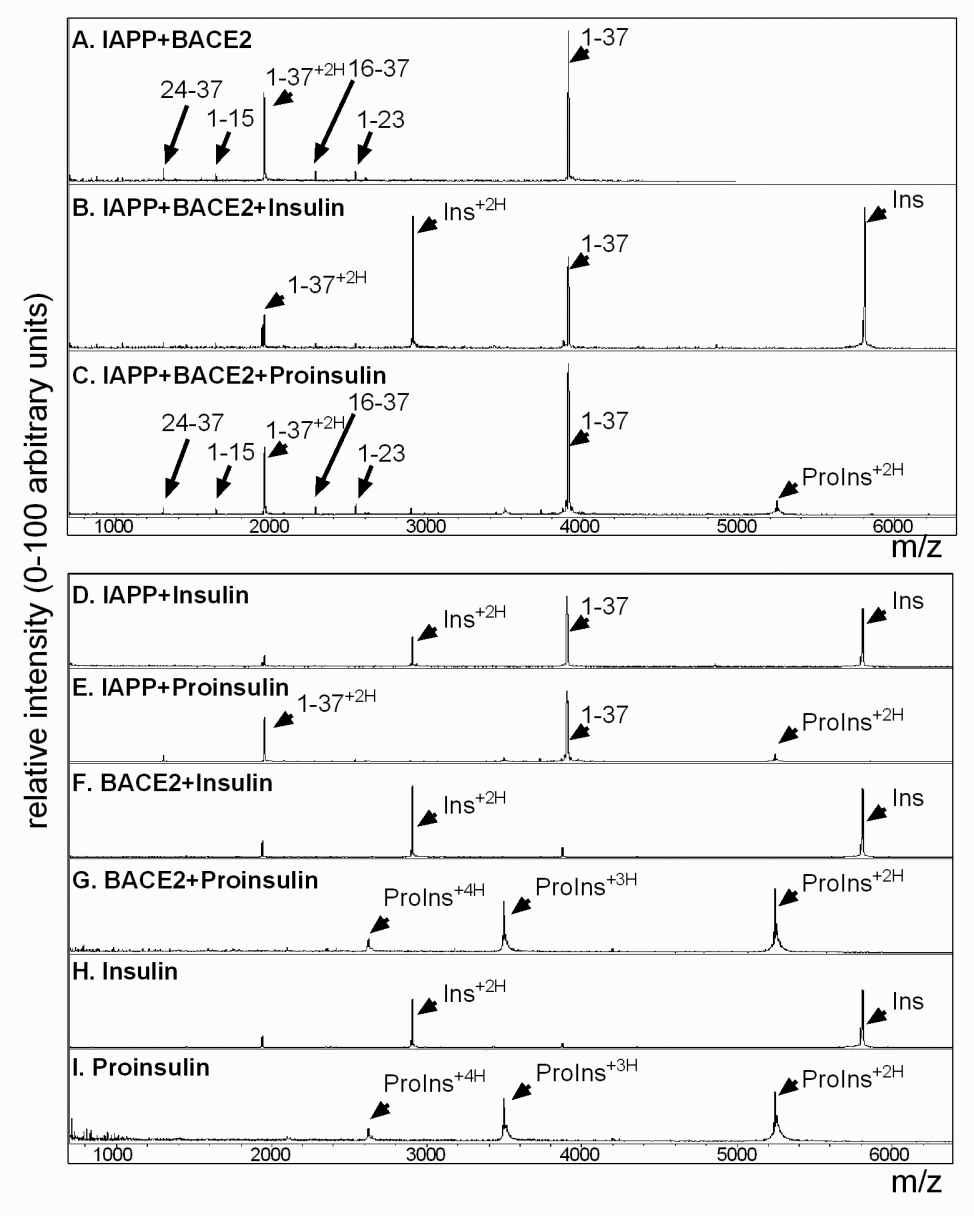
BACE2-mediated proteolytic cleavage of hIAPP is blocked by insulin but not proinsulin. (A) MS analysis of hIAPP incubated with recombinant BACE2; the predicted peaks are indicated: 1–37, 24–37, 1–15, 16–37 and 1–23. (B) Co-incubation with recombinant human insulin blocks BACE2-mediated digestion of hIAPP. (C) Co-incubation with recombinant human proinsulin does not prevent BACE2-mediated digestion of hIAPP. (D) hIAPP plus recombinant human insulin: Single protonated IAPP (m/z 3903) and double protonated IAPP (m/z 1952); single protonated insulin (m/z 5806) and double protonated insulin (m/z 2903). (E) hIAPP plus recombinant human proinsulin; single protonated IAPP (m/z 3903), double protonated IAPP (m/z 1952), double protonated proinsulin (m/z 5250), triple protonated proinsulin (m/z 3495), and quadruple protonated proinsulin (m/z 2625). (F) Recombinant insulin is unchanged by recombinant BACE2. (G) Recombinant proinsulin is unchanged by recombinant BACE2. (H) Recombinant insulin alone and (I) recombinant proinsulin alone. All reactions were performed in PBS, pH 7 for 4 hours at 37°C. Peptide fragment sequences detailed in Table 4.

Human IAPP has two alpha-helical segments, 8–18 and the NFGAIL region, 22–27, which allows hIAPP to adopt α-helical structures to promote dimerization [36]. Structural studies suggest that helical dimerization of hIAPP accelerates fibril formation but that insulin can bind to hIAPP, thus blocking both hIAPP homodimerization and subsequent fibril formation [8, 9]. Accordingly, under homeostatic conditions *in vivo*, even though the concentration of hIAPP present in secretory granules is at least a thousand times more than what is observed to form fibrils *in vitro* [37, 38], hIAPP does not dimerize or form fibrils. As noted previously, mature insulin is stored in excess of IAPP in the secretory granules of beta-cells [4]. Published studies show a direct association between insulin and IAPP and that insulin actually impairs IAPP dimerization by directly binding to IAPP between residues 8–23 [8, 9, 39]. To determine if insulin can block BACE2 enzymatic digestion of hIAPP *in vitro*, insulin was added to mature hIAPP peptide prior to the addition of BACE2 (Fig 5 and Table 4). MS analysis shows that the peaks generated by BACE2 digestion were reduced when insulin was added to hIAPP (Fig 5A and 5B). To assess whether this was a non-specific effect due to the presence of exogenous protein, proinsulin was used as a control. Published BIAcore analysis showed that proinsulin, in contrast to insulin, does not bind well to hIAPP [9]. As predicted, MS revealed no difference to BACE2-mediated digestion of hIAPP with or without proinsulin (Fig 5C). Controls for each of these conditions are shown (Fig 5D–5I).

**Table 4:** Key for Fig 5.

m/z	Description	Sequence
3903	hIAPP 1–37, H^+^	KCNTATCATQRLANFLVHSSNNFGAILSSTNVGSNTY
1952	hIAPP 1–37, 2H^+^	KCNTATCATQRLANFLVHSSNNFGAILSSTNVGSNTY
1382	hIAPP 24–37	GAILSSTNVGSNTY
1639	hIAPP 1–15	KCNTATCATQRLANF
2281	hIAPP 16–37	LVHSSNNFGAILSSTNVGSNTY
2537	hIAPP 1–23	KCNTATCATQRLANFLVHSSNNF
5806	hInsulin, +H	
2903	hInsulin, +2H	
5250	hProinsulin, +2H	
3494	hProinsulin, +3H	
2625	hProinsulin, +4H	

### BACE2 modulates human IAPP fibrillation in a dose dependent manner

Having established that hIAPP is a substrate for BACE2 we next evaluated whether BACE2 activity could modulate fibril formation of hIAPP. Numerous hIAPP-derived peptides are capable of forming amyloid-like fibrils with distinct morphologies [5, 40–42]. However, structural analysis suggests hIAPP adopts transient α-helical structures at residues 8–18 and 22–27 that modulate dimerization [8]. While the N-terminal domain of hIAPP, including residue F15, appears to modulate the kinetics of fibrillation [33], several investigators report that, under physiological conditions, segment 22–29; NFGAILSS, is critical for amyloidogenicity [32, 33, 43, 44] and segments 21–27; NNFGAIL, and 28–33; SSTVNG, contribute to the most common polymorph within the steric zipper motif [36]. Furthermore, whereas peptide 23–27; FGAIL, is the shortest fibrillogenic sequence of hIAPP, peptide 24–27; GAIL, exhibits no amyloidogenecity [44], also suggesting that cleavage at F23 could significantly affect fibril formation. Thus, we predicted both recombinant BACE2 and BACE1 would delay the kinetics of fibril formation due to cleavage at F15, but that BACE2 would have a more profound impact overall due to additional cleavage of hIAPP at F23.

**Fig 6:**
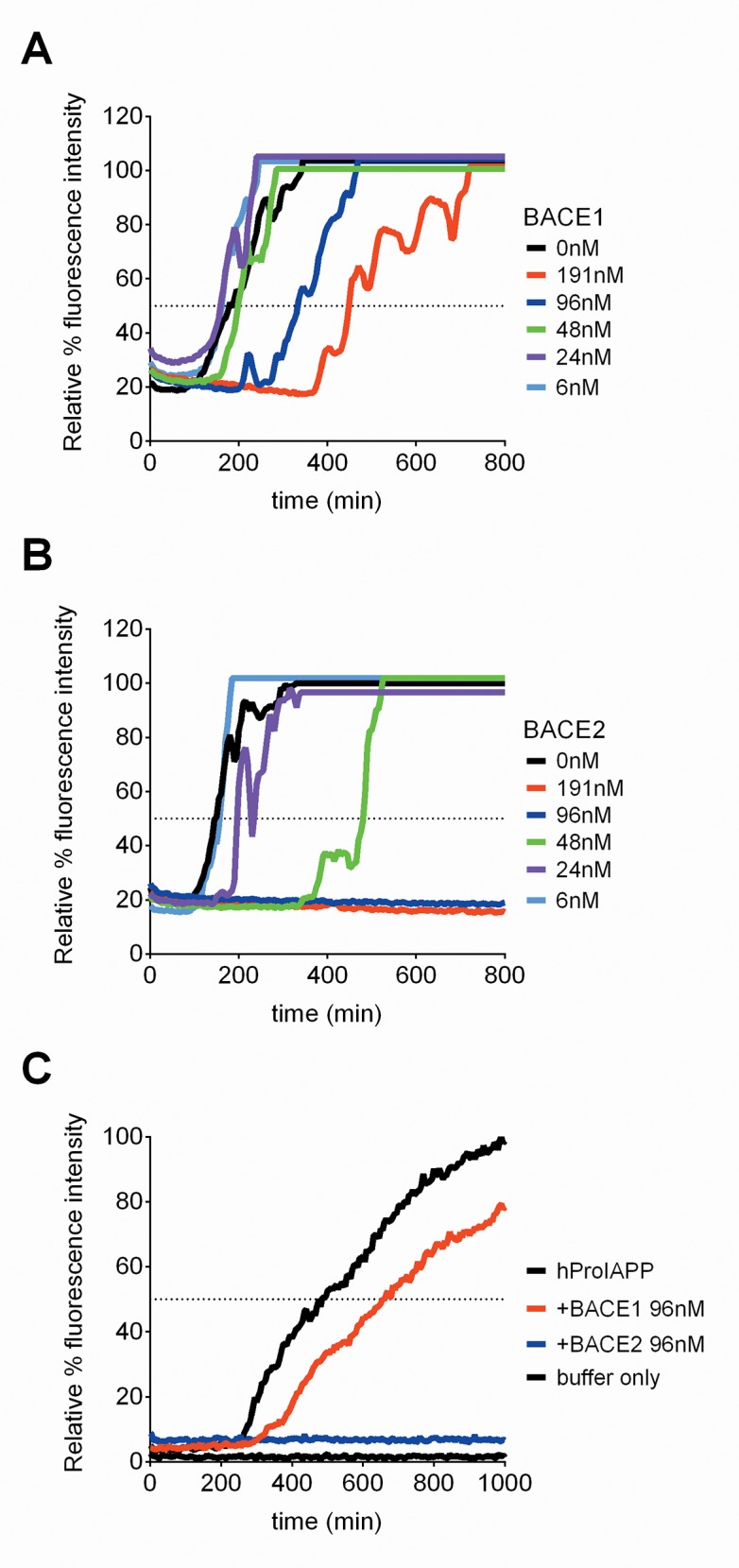
BACE2 modulates hIAPP fibrillation. The Thioflavin T graph demonstrates the time needed (x-axis) to reach 50% fibrillation (dotted black line) of human IAPP (solid black line) as depicted by relative % fluorescence intensity (y-axis). (A) Addition of recombinant BACE1 (6nM-191nM) slows the kinetics of hIAPP fibrillation in a dose-dependent manner. (B) Addition of recombinant BACE2 (6nM-191nM) blocks the kinetics of hIAPP fibrillation in a dose-dependent manner. (C) Addition of recombinant BACE1 (96nM, red line) slows the kinetics of hProIAPP fibrillation, whereas recombinant BACE2 (96nM, blue line) inhibits hProIAPP fibrillation. Lines indicate the average of the triplicate values for each condition.

The kinetics of hIAPP fibril formation as a function of increasing recombinant BACE concentration was determined *in vitro* via incorporation of Thioflavin T (ThT), a spectroscopic dye whose fluorometric properties change upon binding to β-sheet rich amyloid fibrils [45]. The addition of recombinant BACE1 to hIAPP in the ThT assay showed a dose-dependent delay in the rate of hIAPP fibril formation (Fig 6A), consistent with the loss of a portion of the N-terminal helical domain at F15 [8]. When BACE2 was added to hIAPP in the ThT assay, however, a more robust impairment of fibril formation was observed, including complete inhibition of aggregation at high concentrations over the duration of the experiment (Fig 6B). This result is consistent with not only disruption of the N-terminal helical domain of hIAPP at F15 but also obstruction of the FGAILSS region by BACE2-mediated cleavage at F23. Although several cleavage products of hIAPP are amyloidogenic themselves, the rate of aggregation in the context of the ThT assay is dominated by the most amyloidogenic species [46]. Upon cleavage, the C-terminal portion would remain the most aggregation prone and the kinetics would be a function of this segment, as opposed to the N-terminal segment. Thus, while both fragments can form fibrils, the rates of aggregation are vastly different. Similar to the MS assays, these hIAPP findings were repeated with commercial mouse recombinant enzymes and we observed similar trends with both the BACE2 and BACE1 enzymes, including an observed lack of ThT fluorescence at the highest BACE2 concentration (data not shown).

Studies suggest that aberrant or unprocessed ProIAPP may also contribute to IAPP-based amyloidosis [47–49]. Although the kinetics of ProIAPP fibril formation is slower than mature hIAPP, in this ThT system fibrillation of hProIAPP was modulated similarly: the addition of BACE1 delayed fibrillation whereas the addition of BACE2 resulted in complete inhibition (Fig 6C).

### BACE2 modulates hIAPP protein levels in cells

**Fig 7:**
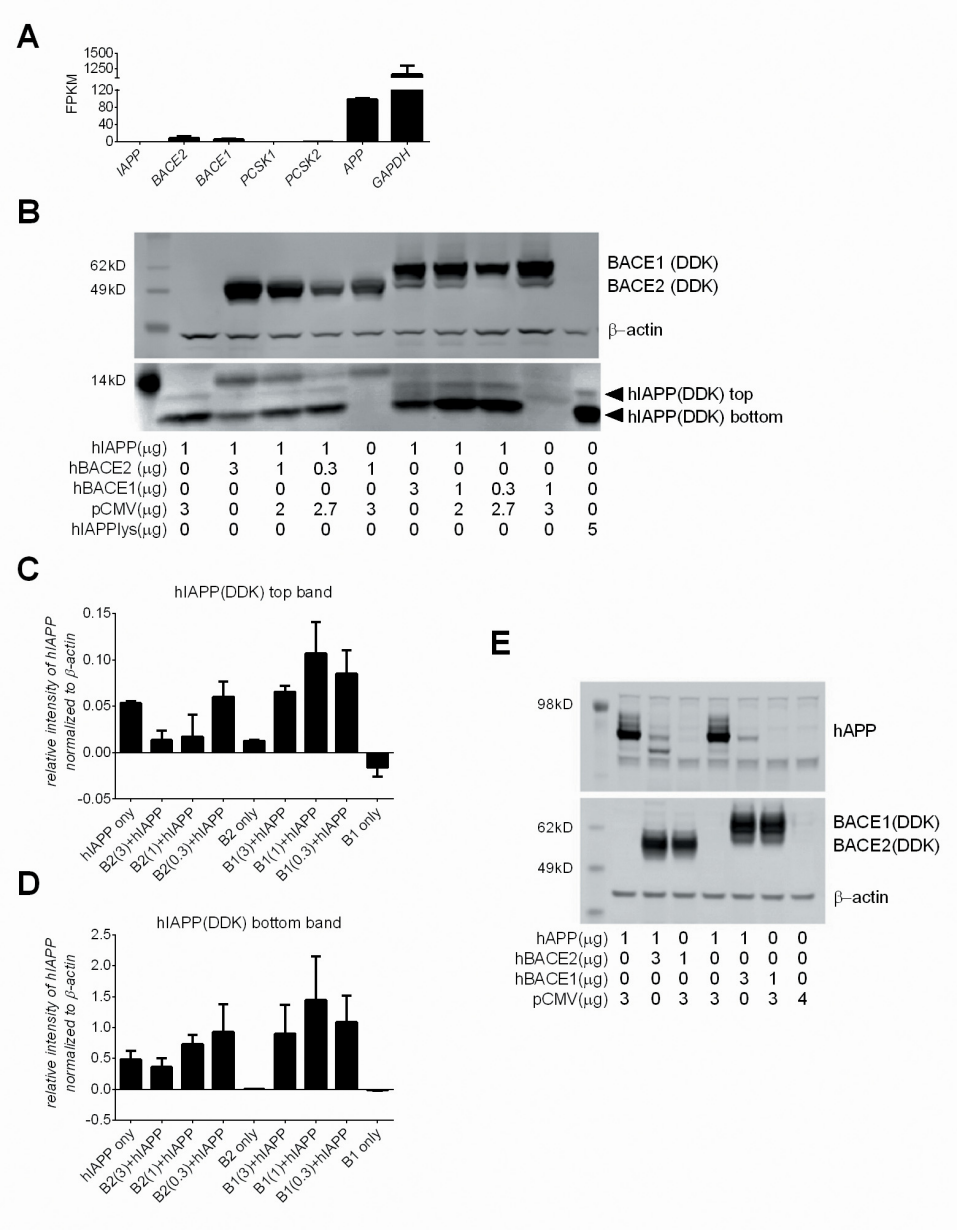
BACE2 modulates human IAPP protein levels HEK293 cells. (A) RNAseq FPKM values for basal expression of human *IAPP*, *BACE2*, *BACE1*, *PCSK1*, *PCSK2* and *APP* in HEK293 cells. (B) HEK293 cells co-transfected with, or without, hIAPP plasmid DNA (1μg) and varying concentrations of hBACE2 plasmid DNA, hBACE1 plasmid DNA, or empty pCMV DNA, the latter to normalize DNA concentrations across conditions. A commercial source of Myc-DDK tagged hIAPP lysate (5 μg, last lane) was used to confirm identification of the hIAPP bands. The relative intensity of the top hIAPP band (C) and bottom hIAPP band (D) for each condition, as detected by anti-DDK and normalized to β-actin. Results are averaged from two separate experiments and the standard deviation is shown. (E) HEK293 cells co-transfected with hAPP, hBACE2, hBACE1 or empty pCMV DNA.

The studies presented so far show that hIAPP peptide is a substrate for BACE2 and that recombinant BACE2 protein significantly impairs hIAPP fibril formation by cleaving the peptide at two distinct sites. We next assessed whether BACE2 can modulate hIAPP protein in a cellular setting. Human embryonic kidney (HEK) 293 cells were chosen for the initial co-transfection studies as, except for human *APP*, low-to-undetectable endogenous expression of human *IAPP*, *BACE2*, *BACE1*, or the IAPP processing convertases *PC2* and *PC1/3*, could be detected by RNA-Seq (Fig 7A). Transfection of HEK293 cells with Myc-DDK tagged hIAPP resulted in the detection of two bands: a faint top band, ~12kD, and a more pronounced bottom band, ~10kD (Fig 7B). There is an additional band, ~14Kd, also present in BACE2 transfected cells, which may represent a nonspecific protein and not hIAPP. Co-transfection of HEK293 cells with Myc-DDK tagged hIAPP plasmid DNA and Myc-DDK tagged hBACE2 plasmid DNA resulted in a reproducible dose-dependent decrease in both the top and bottom hIAPP bands, reaching more than a two-fold reduction in the top band at the highest concentration of hBACE2 tested (Fig 7B–7D). In contrast, co-transfection of HEK293 cells with both hIAPP and Myc-DDK tagged hBACE1 plasmid DNA did not diminish the intensity of either the top or bottom hIAPP protein bands. To assure correct detection of hIAPP-specific bands, a commercial Myc-DDK tagged hIAPP HEK293T cell lysate (Origene) was used as a positive control. The lack of PC2 or PC1/3 in this system suggests that the top band may represent the unprocessed form of hIAPP. Due to differential processing which may occur under the co-transfection conditions, as suggested by the mass spectrometry studies, the bottom band could represent a variety of processed and aggregated species of hIAPP and mature hIAPP. The relative intensity of both the top and bottom hIAPP bands, normalized to β-actin expression, for each condition is shown (Fig 7C and 7D).

To validate our system and confirm the functional activity of the enzymes in this context, HEK293 cells were co-transfected with human amyloid polypeptide (APP) plasmid DNA and either hBACE2 or hBACE1 plasmid DNA. Similar to that observed with hIAPP, hBACE2 co-transfection effectively reduced hAPP protein levels (Fig 7*E*). Contrary to the lack of effect of hBACE1 had on hIAPP, hAPP protein levels were dramatically reduced by hBACE1 co-transfection (Fig 7E).

**Fig 8:**
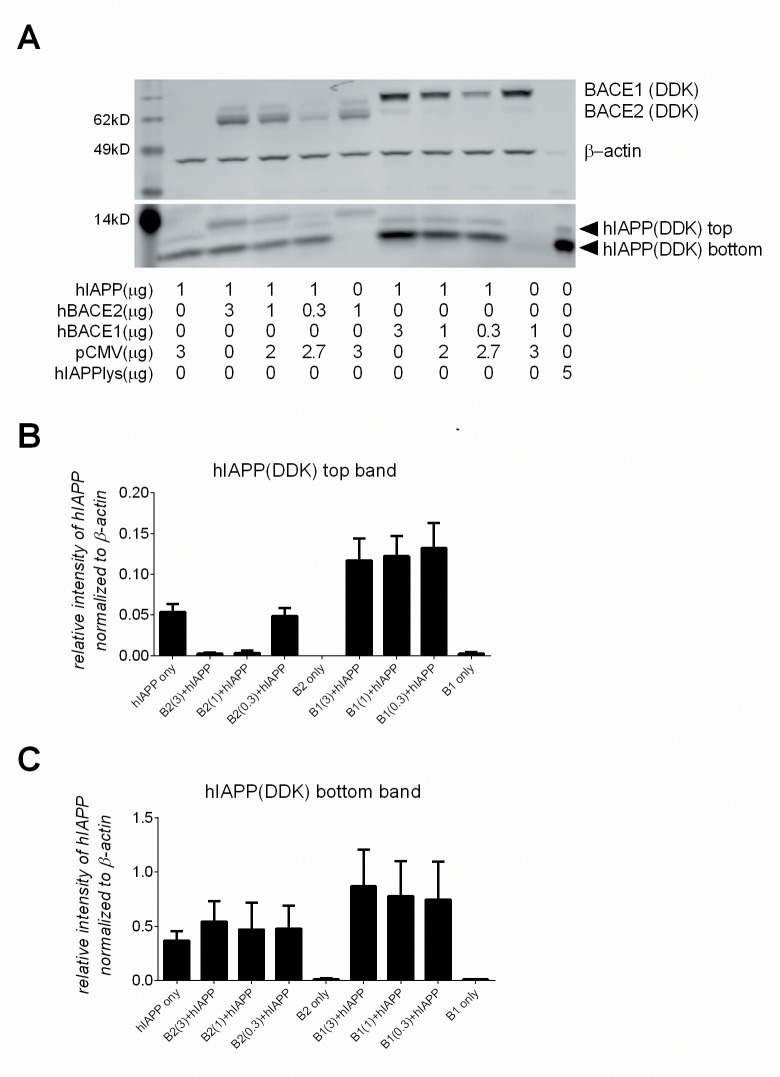
BACE2 modulates human IAPP protein levels in pancreatic beta-cells. (A) βTC3 cells co-transfected with, or without, hIAPP plasmid DNA (1μg) and varying concentrations of hBACE2 plasmid DNA, hBACE1 plasmid DNA, or empty pCMV DNA, the latter to normalize DNA concentrations across conditions. A commercial source of Myc-DDK tagged hIAPP lysate (5 μg, last lane) was used to confirm identification of the hIAPP bands. The relative intensity of the top hIAPP band (B) and bottom hIAPP band (C) for each condition, as detected by anti-DDK and normalized to β-actin. Results are averaged from five separate experiments and the standard error is shown.

To determine if these findings translate to the context of pancreatic beta-cells, complete with all the machinery and enzymes distinct to IAPP processing, mouse βTC3 cells were used for similar co-transfection studies. Co-transfection of βTC3 cells revealed a similar phenomenon to that of HEK293 cells; a dramatic loss of the top hIAPP band (~12kD) with co-transfection of hBACE2, and no change in the top band with co-transfection of hBACE1 (Fig 8A). The relative intensity of both the top and bottom hIAPP bands, normalized to β-actin expression, for each condition is shown (Fig 8B and 8C). Despite the endogenous expression of the proconvertases and beta secretases in these cells, over-expression of hBACE2 effectively reduced the top hIAPP band, while over-expression of hBACE1 stabilized protein levels (Fig 8B and 8C). In contrast to HEK293 cells, the bottom hIAPP band in βTC3 cells was less affected by the presence of hBACE2. Since IAPP is predominantly expressed by beta-cells as the pro-IAPP form [5], this observation may reflect the net product of several processed species of hIAPP, by the endogenous proconvertases, BACE2 or BACE1, or the exogenous enzymes (Fig 8C). Altogether, the co-transfection studies further support hIAPP as a BACE2 substrate and suggest translation of this enzymatic process at a cellular level.

### BACE2 inhibition does not induce beta-cell proliferation in vivo

While investigating the effect of BACE2 on hIAPP fibrillation, we considered identification of this novel role for BACE2 in beta-cells and hyperamylinaemia may be controversial due to reports of BACE2 inhibition as a therapy for T2D [21, 24, 50]. Specifically, investigators identified TMEM27, a beta-cell-specific transmembrane protein, as susceptible to cleavage by BACE2 [21]. Using an *ob/ob* diabetic mouse model, the investigators showed that chronic treatment with a small molecule inhibitor (SMI) for BACE2 prevented cleavage of the extracellular domain of TMEM27, resulting in improved glucose tolerance and the proliferative capacity of beta-cells. Thus, we performed a similar SMI study to that published [21] using both the relatively non-selective SMI described in the publication, Compound J, and another well characterized non-selective BACE SMI, Compound 15 [29].

**Fig 9:**
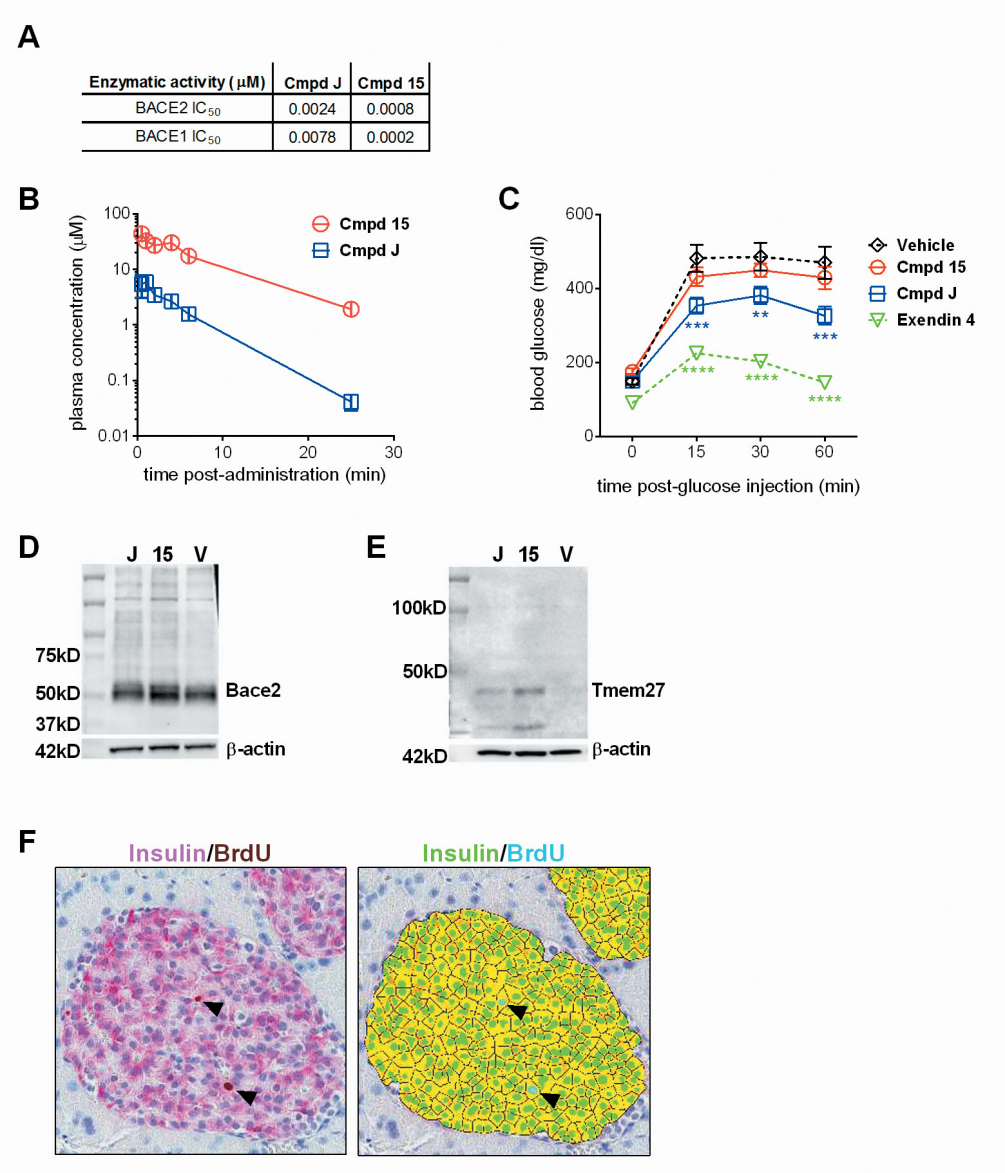
Evaluation of BACE inhibitors, Compound J and Compound 15, in B6.Cg-Lep^ob^/J mice. (A) The IC_50_s (μM) for Compound J and Compound 15. (B) Plasma clearance of Compound J and Compound 15 after a single oral gavage administration at 30mg/kg in 8–10 week old male B6.Cg-Lep^ob/J^ mice, n = 2 per time point. (C) Blood glucose levels before (0 minutes) and after an intraperitoneal glucose injection (10%/kg body weight) in B6.Cg-Lep^ob/J^ mice treated daily for 14 days with vehicle (black diamond, n = 14), Compound 15 (red circle, n = 13), Compound J (blue square, n = 13), or Exendin 4 (green triangle, n = 14). Asterisks indicate statistical significance based on 2-way ANOVA analysis, ** = 0.007, ***≤ 0.0007, **** < 0.0001. Target coverage analysis for BACE2 (D) and Tmem27 (E) on islets isolated from B6.Cg-Lep^ob/J^ mice treated with a single dose (30mg/kg, by oral gavage) of Compound J, Compound 15, or vehicle; n = 2 per group and islets were pooled for protein analysis. (F) One day before harvest, animals were injected with BrdU and pulsed for 24 hours prior to termination. Pancreas tissue was collected, stained for insulin and BrdU expression, and evaluated for several parameters to quantitate beta-cell proliferation. The image on the left shows a representative islet stained for insulin (Vulcan Red) and BrdU (DAB); the arrows point to Insulin^+^BrdU^+^ cells. The corresponding image demonstrates the morphometric analysis applied to quantitate proliferating beta-cells: individual insulin^+^ cells (green), insulin^+^BrdU^+^ cells (blue, indicated by arrows).

First, using a fluorescence resonance energy transfer (FRET) based inhibitor activity assay, the IC_50_ for BACE2 and BACE1, respectively, was determined for Compounds J and 15 (Fig 9A); the selectivity of Compound J for BACE2:BACE1 was similar to that described [21]. In the reported study, Compound J was administered in gelatin by intraperitoneal injection. Our formulation analysis determined dissolving the compounds in 10% HPbCD in water, pH2.2 with HCl was optimal for complete solubilization of both compounds (data not shown). Therefore, we chose this formulation for our studies. Due to the acidity of the solution, we also chose oral gavage dosing of the compounds to avoid injection site irritation. *In vivo* pharmacokinetic analysis for Compounds J and 15, performed in parallel, showed a profile very similar to that reported for J [21] and relatively stronger pharmacokinetics for Compound 15 (Fig 9B).

For the chronic dosing study, treatment with Exendin 4 was included as a positive control as it has been well documented to induce beta-cell proliferation in a variety of diabetic mouse models [51]. After 14 days of chronic dosing in the B6.V-Lep^ob^/J mouse model, we observed a modest improvement in glucose disposal with Compound J and no significant change with Compound 15 (Fig 9C). To confirm the SMI compounds stabilized BACE2 and prevented TMEM27 cleavage, as described previously [21], additional mice were dosed with each compound, islets were isolated, pooled, and cell lysates prepared. Western Blot analysis confirmed *in vivo* stabilization of BACE2 (Fig 9D) and expression of full-length TMEM27 (Fig 9E) with both compounds, indicating *in vivo* target coverage. However, extensive analysis on pancreas tissue collected after 3 weeks of chronic dosing (Fig 9F) did not reveal a difference in beta-cell numbers in either Compound J- or Compound 15-treated mice, while Exendin 4-treated mice exhibited the predicted increase in beta-cell proliferation (Table 5). Thus, despite stabilization of full-length TMEM27, we were not able to reproduce the described *in vivo* effect of BACE2 inhibition on beta-cell proliferation.

**Table 5:** Morphometric analysis of pancreas tissue from the chronic dosing study.

	Treatment			
Parameters	Vehicle	Compound J	Compound 15	Exendin 4
Animals evaluated per group	12	11	13	14
Pancreas area (μ^2^)	9.7e7 ± 3.9e6	9e7 ± 5.2e6	9.2e7 ± 5.1e6	9.5e7± 5.05e6
Total insulin^+^ area (μ^2^)	2.2e6 ±1.5e5	2.3e6 ± 2.4e5	2.3e6 ± 1.9e5	3.7e6 ± 9.6e5
Insulin^+^/pancreas area (μ^2^)	2.3 ± 0.2	2.5 ± 0.1	2.5 ± 0.2	3.9 ± 1.0
Insulin^+^ cells	17515 ± 1010	17781 ± 1756	19031 ± 1618	21543 ± 1353
BrdU^+^Insulin^+^ cells	64 ± 8	66 ± 9	57 ± 7	85 ± 9
BrdU^+^Insulin^+^ cells/islet	0.25 ± 0.02	0.23 ± 0.02	0.23 ± 0.02	0.27 ± 0.03
Total number of islets	251 ± 14	279 ± 19	245 ± 13	317 ± 16
Average islet size (μ^2^)	8700 ± 315	8168 ± 459	9347 ± 468	11704 ± 2854

For each animal, 5 sections, ≥ 300 microns apart, were evaluated and the sum calculated. Values represent the averages of those sums for every animal per cohort ± standard error. Proliferating cells were identified by BrdU staining.

## Discussion

The data presented herein establish hIAPP is a substrate for BACE2 and that the resulting proteolysis of this interaction can significantly modulate hIAPP fibril formation. Proteolytic cleavage of hIAPP by BACE2 occurs at the phenylalanine residues at positions 15 and 23; BACE1 can also cleave hIAPP but only at position F15. BACE1-mediated cleavage significantly prolonged the lag phase observed in hIAPP fibril formation; however, the additional cleavage at position F23 by BACE2 appears to modulate hIAPP fibril formation to an even greater extent. These observations are consistent with numerous studies exploring distinct regions and residues of IAPP and their specific roles in fibril formation. The capacity of BACE2 to cleave super-optimal concentrations of hIAPP *in vitro* and modulate fibrillogenesis suggests a potential new therapeutic approach to overcoming amyloidosis associated with T2D. The data suggest a therapeutic approach promoting cleavage of hIAPP at the F15 and F23 sites may lower the rate of aggregation *in vivo*, which could provide therapeutic benefit.

The proposed proteolytic action of BACE2 on hIAPP was also assessed in a cellular context. While BACE1 co-transfection did not result in any detectable degradation of hIAPP, BACE2 co-transfection markedly reduced the detectable level of hIAPP protein in both HEK293 cells and βTC3 cells. An assay that clearly demonstrates consistency in the precursor and the summation of the proteolyzed products would have been ideal; however there are numerous technical challenges when working with hIAPP, including reliable and sensitive detection, possible aggregation of proteolyzed products, and, particularly in this circumstance, very low molecular weight products. We found using an anti-DDK antibody combined with an 18% gel to be the most sensitive and reliable way to monitor hIAPP in these assays, however this meant that only products containing the tag were captured. Furthermore, it is possible that, once cleaved, peptide fragments are further degraded, adding to the loss of detectable product; this would be akin to the identification of BACE2 as an avid Aβ degrading protease [17]. We recognize there could be an alternative explanation for the loss of hIAPP when co-transfected with BACE2, and that the cell assays performed do not directly demonstrate cleavage, however, the reproducible and dose-dependent loss of the top hIAPP band supports the defined biochemical reactions included in the MS data clearly depicting both input and product, and thus, together with the ThT data, collectively support hIAPP as a BACE2 substrate.

A therapy that can impair hIAPP dimerization, and thereby modulate oligomerization, may be beneficial beyond the context of the pancreas. Relevant to our studies and the shared capacity of BACE2 to proteolytically cleave IAPP and Aβ, Jackson *et al* described identifying IAPP deposition in the temporal lobe gray matter, blood vessels and perivascular spaces of T2D patients and found mixed IAPP-Aβ deposits in the blood vessels and brain parenchyma of late onset AD patients [52]. IAPP-Aβ cross-amyloid interaction has also been observed in the islet amyloid aggregates found in T2D patients [53]. Studies by Andreetto et al [54] led to the identification of “hot regions” of the IAPP and Aβ interface that are high affinity ligands of both IAPP and Aβ. Systematic evaluation of IAPP peptide regions determined IAPP (8–18) and IAPP (22–28) sequences as self-interaction and cross-interaction hot regions. Based on our findings that BACE2 proteolytically cleaves hIAPP at residues F15 and F23, one may predict BACE2 therapy as means for disrupting both IAPP-IAPP and IAPP-Aβ interactions at a molecular level and, in so doing impede homo- and hetero-amyloidosis.

Although our findings support a positive role for BACE2 activity in beta-cells, we felt it necessary to address the reported findings of a negative role for BACE2 in beta-cell proliferation and glucose homeostasis, namely by proteolytic cleavage of the extracellular domain of the beta-cell-specific transmembrane protein, TMEM27 [21]. Comparing the reported SMI, Compound J, to another well-characterized SMI, Compound 15, we did not observe any effect of these SMIs on beta-cell proliferation in diabetic mice despite target coverage. The lack of testing of these SMIs in BACE2-deficient mice in both our study and that published [21] raises the question of possible off-target effects and/or modulation of other BACE2 substrates in pancreatic beta-cells, such as SEZ6L2 [24]. Our findings are consistent with those presented as an abstract by researchers at Novartis; the potent non-selective BACE1/BACE2 inhibitor, SMI NB-360, did not improve hyperglycemia, glucose tolerance or increase beta-cell mass after chronic treatment in diabetic *ob/ob* mice [55]. Furthermore, the lack of expression differences between *Tmem27* on islets from healthy versus T2D subjects [56] and the loss of beta-cells during T2D disease progression poses the question of relevance of BACE2-mediated inhibition of TMEM27 cleavage as a therapy in humans. Due to the lack of amyloidosis in rodents, the possible effect of a BACE2 inhibitor on IAPP would be undetectable in this model. Nonetheless, the data highlight the existence of multiple BACE2 substrates in pancreatic beta-cells and signify a rationale to further investigate BACE2-substrate interactions and how they translate to humans and the disease process.

The phenotypes associated with hIAPP-mediated amyloidosis are extensive and the findings from our studies suggest a multidimensional purpose for targeting hIAPP therapeutically. Our findings present a new basis to further investigate BACE2-mediated proteolytic cleavage of hIAPP *in vivo* and ascertain whether there is a translational benefit to BACE2 therapy for T2D. Although ridding the body of preformed aggregated plaques is a technical challenge yet to be solved, our data suggest cleavage of hIAPP at the F15 and F23 residues may reduce the burdening effects of hyperamylinaemia that manifests in T2D patients.
